# Editorial for Special Issue “Antimicrobial Treatment of Lower Respiratory Tract Infections”

**DOI:** 10.3390/antibiotics14030232

**Published:** 2025-02-25

**Authors:** Charalampos D. Moschopoulos, Anastasia Kotanidou, Sotirios Tsiodras, Paraskevi C. Fragkou

**Affiliations:** 1 Fourth Department of Internal Medicine, School of Medicine, Attikon University Hospital, National and Kapodistrian University of Athens, 12462 Athens, Greece; bmosxop@yahoo.gr (C.D.M.); sotirios.tsiodras@gmail.com (S.T.); 2 First Department of Critical Care Medicine and Pulmonary Services, School of Medicine, Evangelismos Hospital, National and Kapodistrian University of Athens, 10676 Athens, Greece; akotanid@med.uoa.gr; 3 Internal Medicine Department, Aegli Medical Center, 19003 Attika, Greece

Lower respiratory tract infections (LRTIs) are highly prevalent, and severe LRTIs are associated with significant mortality and morbidity worldwide [1]. Pneumonia remains among the main causes of morbidity and mortality in adults and represents the leading cause of death in children under the age of five [2,3]. Pneumonia, specifically severe community-acquired pneumonia (CAP) and nosocomial pneumonia, as well as other types of severe LRTIs, are associated with huge socioeconomic and healthcare costs. Antimicrobials remain the mainstay of bacterial LRTI treatment, although their use in viral LRTIs may be associated with significant side effects. Prompt initiation of pathogen-specific antimicrobials is crucial for improving the prognosis and outcome of LRTIs and plays a pivotal role in reducing their global healthcare and socioeconomic burden. The present issue provides insights into treating CAP, healthcare-associated pneumonia (HAP), and pneumonia due to multidrug-resistant organisms (MDROs), and using biomarkers and imaging in primary care to reduce antibiotic prescriptions.

Primary care physicians are often the first point of contact for patients presenting with symptoms of pneumonia, such as fever, cough, and shortness of breath. Primary care is critical in managing CAP, particularly prevention, triage, and antimicrobial stewardship. Evidence supports the use of procalcitonin-guided antibiotic prescriptions for CAP in order to restrict inappropriate antibiotic use in the primary care setting. However, this guidance may often be overruled by general practitioners. Knusli et al. (Contribution 1). interrogated the factors associated with the overruling of procalcitonin-guided treatment and found that socio-demographic characteristics of physicians, such as an urban setting and years of experience, significantly impacted this practice. Another useful tool to support physicians’ decisions on antibiotic prescription is the chest X-ray (CXR).

Carlsson et al. (Contribution 2) performed a register-based study to find factors associated with antibiotic prescription in patients with no findings in their CXR. Residents were prescribed antibiotics more often than interns or specialists, but no other potential causes were identified. Moreover, Fischer et al. (Contribution 3) investigated whether clinical indices, such as oxygen saturation, could rule out pneumonia and avoid unnecessary CXRs. They showed that incorporating pulse oximetry into a simple clinical decision rule could effectively reduce the likelihood of pneumonia to an acceptable level, thereby minimizing the need for unnecessary imaging. Finally, Gancitano et al. Contribution 4) performed a systematic review and meta-analysis to examine the effect of Echinacea in reducing antibiotic prescription by preventing respiratory tract infections. They showed that Echinacea supplements effectively reduced monthly RTI occurrence, recurrent RTI, RTO complications, and the need for antibiotic therapy, with a decrease in days of antibiotic treatment by 70%.

Third-generation cephalosporins and beta-lactam/beta-lactamase inhibitor combinations are among the most commonly used antibiotics for the initial treatment of CAP, especially in the presence of comorbidities or need for hospitalization [4]. Beta-lactam/beta-lactamase inhibitor combinations are generally considered more appropriate when oral anaerobes come into play (e.g., in aspiration pneumonia), but comparative evidence between the two antibiotic regimens is lacking. Kato et al. (Contribution 5) conducted a systematic review and meta-analysis to compare the effect of ceftriaxone and ampicillin/sulbactam as initial treatment of CAP on mortality and clinical cure, and their findings indicated no significant differences in the mortality and clinical cure rates between the two groups. Further, Vallianou et al. (Contribution 6) discussed the significance of lung microbiome in the management and treatment of aspiration pneumonia, especially with the use of novel metagenomic next-generation sequencing to promptly identify the causative agent(s) and genetic antimicrobial resistance.

In the pediatric population, antibiotic selection for CAP is particularly important in order to avoid antibiotic-related complications and the emergence of antimicrobial resistance. Puzz et al. (Contribution 7) evaluated an antimicrobial stewardship (AMS) intervention to optimize antibiotic selection and treatment duration in hospitalized children with CAP in accordance with current guidelines. They found that AMS led to a significant reduction in the use of ceftriaxone and a corresponding increase in the use of ampicillin, as well as a reduction in treatment duration from 10 days (pre-AMS) to 8 days (post-AMS).

In the context of HAP, antimicrobial resistance and difficult-to-treat infections are increasingly becoming the primary focus across the globe. A debatable point is whether piperacillin/tazobactam could be used as an alternative to carbapenems for extended-spectrum beta-lactamase (ESBL)-producing microorganisms. To answer this, Zha et al. (Contribution 8) conducted a retrospective cohort study to compare the two antibiotic regimens in patients with HAP caused by ESBL-producing *Klebsiella pneumoniae* that was sensitive to both antibiotics. This study found no significant differences in 28 day mortality, clinical, and microbiological cure rates between the groups, showing that piperacillin/tazobactam could be an effective alternative to carbapenems for these infections when the MICs are ≤8 mg/L. 

Moreover, difficult-to-treat infections include those caused by carbapenem-resistant bacteria, particularly *Acinetobacter baumanii* and *Klebsiella pneumoniae*. Zha et al. (Contribution 9) examine the benefits of adding polymyxin B to high-dose tigecycline to treat pneumonia caused by these microorganisms. Their propensity score-matched cohort study found that combination therapy was not associated with better clinical outcomes and showed similar 14-day mortality, clinical and microbiological cure, and rate of nephrotoxicity with high-dose tigecycline alone. In addition, Almyroudi et al. (Contribution 10) presented an up-to-date review of novel antibiotics licensed for the treatment of Gram-negative HAP, with a special focus on antibiotics targeting MDROs.

Another challenging aspect of pneumonia management is the treatment of complications, such as empyema. Shiroshita et al. (Contribution 11) investigated whether empirical anti-pseudomonal antibiotics were warranted in patients with empyema undergoing thoracoscopy. Their analysis showed no extension in time to death and thoracic surgery within 90 days and no reduction in 90-day mortality with the use of empirical anti-pseudomonal treatment, irrespective of the patient’s risk for MDRO infection.

Finally, the optimal duration of antibiotic treatment remains a highly debated topic across various clinical settings, including CAP and HAP. This is comprehensively discussed by Dimopoulou et al. (Contribution 12), who gathered the available evidence and concluded that shorter antibiotic regimens are feasible and effective in selected patient populations, while personalized antimicrobial therapies are imperative to improve patient outcomes.

In conclusion, LRTIs pose a major global health burden, contributing significantly to morbidity, mortality, and healthcare costs. This Special Issue emphasizes the critical role of antimicrobial stewardship, optimized antibiotic selection, and innovative diagnostic tools in improving patient outcomes and reducing unnecessary antibiotic use. Pathogen-specific therapy, careful use of biomarkers, primary care imaging, shorter antibiotic regimens, a multidisciplinary approach integrating personalized antimicrobial therapy, and effective stewardship programs can improve LRTI management across various settings and mitigate their adverse outcomes. Continued research remains crucial in addressing the evolving challenges of resistant pathogens and ensuring optimal patient care.
